# West Nile virus surveillance using sentinel birds: results of eleven years of testing in corvids in a region of northern Italy

**DOI:** 10.3389/fvets.2024.1407271

**Published:** 2024-05-16

**Authors:** Marco Tamba, Paolo Bonilauri, Giorgio Galletti, Gabriele Casadei, Annalisa Santi, Arianna Rossi, Mattia Calzolari

**Affiliations:** Istituto Zooprofilattico Sperimentale della Lombardia e dell’Emilia Romagna, Brescia, Italy

**Keywords:** active surveillance, magpie (*Pica pica*), hooded crow (*Corvus cornix*), *Corvidae*, West Nile virus

## Abstract

The natural transmission cycle of West Nile virus (WNV) involves birds as primary hosts and mosquitoes as vectors, but this virus can spread to mammals, human beings included. Asymptomatic infected donors pose a risk to the safety of blood transfusions and organ transplants, as WNV can be transmitted through these medical procedures. Since 2009, the region of Emilia-Romagna in northern Italy has been implementing an integrated surveillance system in order to detect WNV circulation in the environment at an early stage. Here we report the results of the two components of the surveillance system, the active testing of corvids and humans, and demonstrate that bird surveillance alone improves a surveillance system based solely on human case detection. As WNV risk reduction measures are applied on a provincial basis, we assessed the ability of this surveillance system component to detect virus circulation prior to the notification of the first human case for each province. Overall, 99 epidemic seasons were evaluated as a result of 11 years (2013–2023) of surveillance in the nine provinces of the region. In this period, 22,314 corvids were tested for WNV and 642 (2.9%) were found to be infected. WNV was generally first detected in birds in July, with sample prevalence peaks occurring between August and September. During the same period, 469 autochthonous human cases were notified, about 60% of which were reported in August. WNV was detected 79 times out of the 99 seasons considered. The virus was notified in birds 73 times (92.4%) and 60 times (75.9%) in humans. WNV was first or only notified in birds in 57 seasons (72.1%), while it was first or only notified in humans in 22 seasons (27.8%). Active surveillance in corvids generally allows the detection of WNV before the onset of human cases. Failure of virus detection occurred mainly in seasons where the number of birds tested was low. Our results show that active testing of a minimum of 3.8 corvids per 100 km^2^ provides a satisfactory timeliness in the virus detection, but for early detection of WNV it is crucial to test birds between mid-June and mid-August.

## 1 Introduction

West Nile virus (WNV) is a neurotropic mosquito-borne flavivirus that belongs to the Japanese encephalitis serocomplex group. WNV is considered to be the most widespread arbovirus in the world and has been circulating in Europe for decades, where it has increased significantly in recent years. The natural infection cycle involves mosquitoes, mainly *Culex* spp. and birds, while horses and humans are considered to be dead-end hosts. Most human cases are asymptomatic, however 20–40% of infected individuals present with clinical symptoms ranging from influenza syndrome to encephalitis (1). Asymptomatic infected donors pose a risk to the safety of blood transfusion and solid organ transplantation, as WNV can be transmitted through these medical procedures (2–5). Timely detection of WNV circulation is therefore essential for effective implementation of measures such as safety procedures for blood collected for donation, vector control, and communication to relevant Animal and Public Health authorities and to the general public (6).

In Europe, where WNV appears to have limited or no pathogenicity in birds (7), it is recommended to combine passive surveillance in wild birds with some form of active surveillance (8). The use of sentinel birds has been proposed as an effective strategy for early detection of WNV circulation and identification of affected areas (6, 9). Passeriformes, and especially the *Corvidae* family, are highly susceptible to WNV infection (10–12). Eurasian magpies (*Pica pica*) have been successfully used to detect WNV circulation in endemic areas in France (13), Greece (14), and Spain (15). Several factors make this species and the hooded crow (*Corvus cornix*) good sentinels (13, 15): they are territorial and abundant; they have a wide distribution, including near houses, and they also have a scavenging behavior that favors infection with WNV by the oral route. In addition, because magpies, jays and crows prey on eggs and chicks of songbirds and gamebirds and eat berries or other fruits, they are considered nuisance bird species and are often subject to population control in Europe (16), a type of measure that may be useful for WNV surveillance, as in our case.

WNV was first detected in the Emilia-Romagna region of northern Italy in 2008 (17), and an integrated surveillance system has been in place since 2009 in order to: (1) detect the circulation of WNV in the environment (mosquitoes and birds) early, and (2) reduce the risk of virus transmission through blood donations. WNV is actively monitored in mosquitoes, birds, horses and humans with neurological disease symptoms (18, 19). Over time, the system has proven to be economically sustainable and capable of detecting the virus in the environment prior to the onset of human disease (19, 20).

The mosquito surveillance component of our regional system has already been described elsewhere (19, 21, 22). In this paper, we detail the results of the surveillance system component in relation to active surveillance in corvids, which resulted in the ability to detect virus circulation prior to notification of human WNV infection cases, despite the mosquito surveillance data. We believe that our data will provide useful information for the implementation of a WNV early warning system that can effectively improve and integrate a surveillance system based only on human cases.

## 2 Materials and methods

### 2.1 Survey area

**Figure 1:**
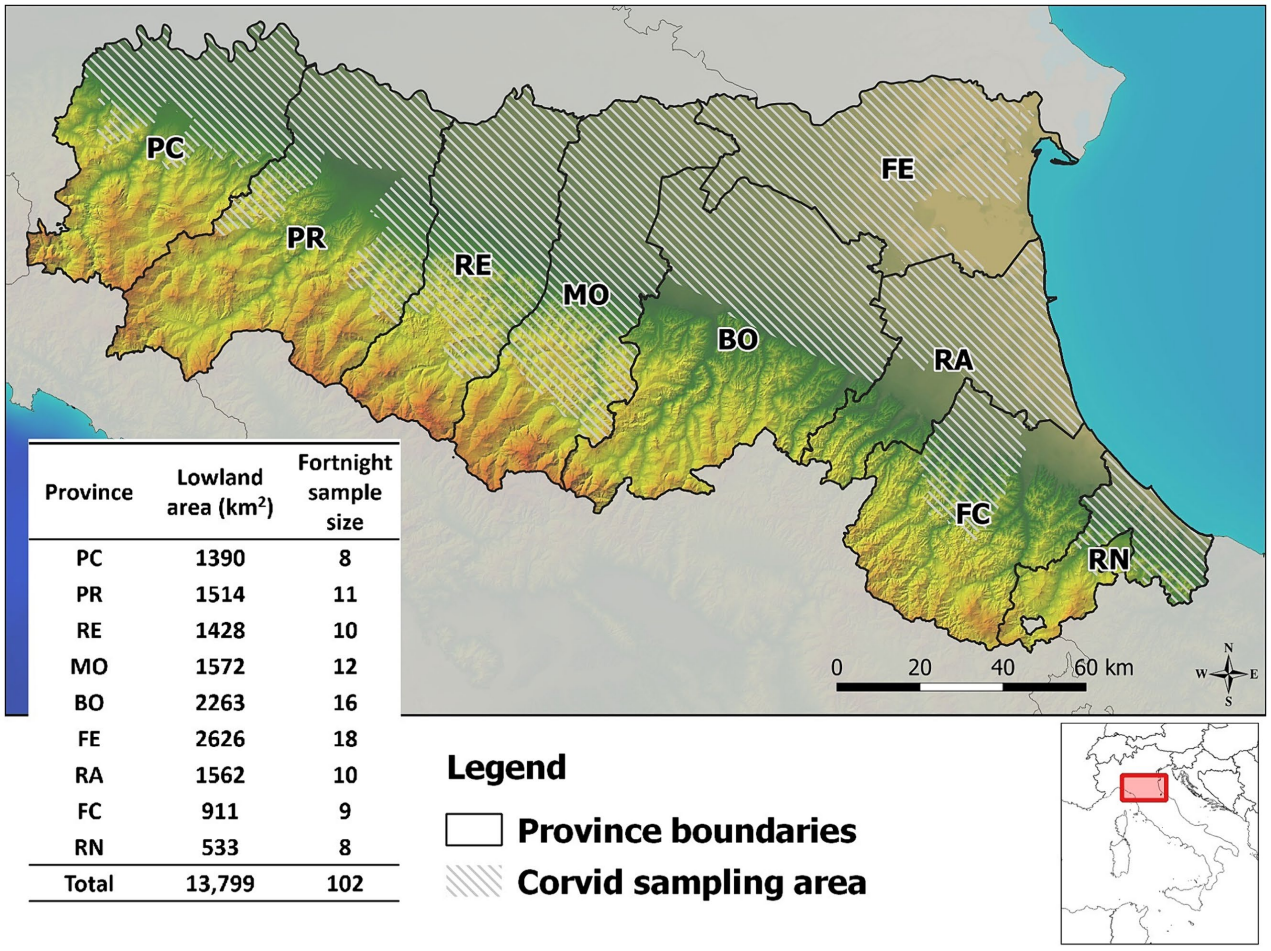
Map of corvid sampling area and sample size provided in the West Nile virus surveillance program in Emilia-Romagna region, 2013–2023. Provinces of Emilia-Romagna region: Piacenza (PC), Parma (PR), Reggio Emilia (RE), Modena (MO), Bologna (BO), Ferrara (FE), Ravenna (RA), Forlì-Cesena (FC), Rimini (RN).

The survey was carried out in the Emilia-Romagna region of northern Italy, which covers an area of 22,509 km^2^, of which about half (13,799 km^2^) is lowland, including the southern part of the Po river valley. The region has a population of about 4.5 million people and is administratively divided into nine provinces, from west to east: Piacenza (PC), Parma (PR), Reggio Emilia (RE), Modena (MO), Bologna (BO), Ferrara (FE), Ravenna (RA), Forlì-Cesena (FC), Rimini (RN). All provinces have lowland areas and are covered by the WNV integrated surveillance program. Birds are sampled only in the lowlands, where the WNV vector population (*Culex pipiens* mosquito) is most abundant (Figure 1).

### 2.2 Sampling and testing of corvids

The Italian national integrated WNV surveillance program requires annual testing of at least 100 sentinel birds every 1,600 km^2^, equivalent to 6.25 birds/100 km^2^, and samples must be collected between April and November of each year (23). As April and November are generally cold months with no mosquito activity, the sampling period in Emilia-Romagna was reduced from 1 May to the end of October. Regional programming requires that bird sampling be divided into two week periods, and that hunting districts should provide a constant flow of samples homogeneously distributed in time and space (Figure 1). Between May and October 2013–2023, 15,632 European magpies (*Pica pica*), 4,670 hooded crows (*Corvus cornix*), and 2012 Eurasian jays (*Garrulus glandarius*) were collected during official pest bird control programs in the Emilia-Romagna region. In all provinces of the region, birds were shot or captured using Larsen traps and killed by trained hunters by cervical dislocation in accordance with the provisions of the national legislation on animal welfare [Council Regulation (EC) 1099/2009]. Sampling was carried out on a voluntary basis under the supervision of the official veterinary services, which ensured rapid delivery of the birds to the laboratory in charge of testing.

From each sampled bird, heart, brain, kidney, and spleen were pooled, mechanically homogenized and tested by real-time PCRs to detect WNV RNA (24–26). Samples that tested positive were also subjected to traditional PCR to obtain amplicons for sequencing. All positive samples were subjected to a pan-flavivirus protocol targeting the NS5 gene (27) and a specific protocol targeting the E gene (28). All tests were performed in the same laboratory (Istituto Zooprofilattico Sperimentale della Lombardia e dell’Emilia Romagna; IZSLER, Reggio Emilia site).

The following data were used for statistical analysis: bird species, sampling date, sampling province, date of delivery to the laboratory, testing start date, test result, date of notification of positive result.

### 2.3 Data on human cases of West Nile disease

WNV infection has been a notifiable disease in Italy since 2007 (5). The human surveillance system requires clinicians to report all possible, probable and confirmed cases of WNV using a modified European case definition that includes neurological symptoms among the clinical criteria (23). A possible case is defined as any person fulfilling the clinical criteria of fever ≥38.5° C, and at least one of the following: viral encephalitis, viral meningitis, polyradiculoneuritis (a condition similar to Guillain–Barré syndrome), and acute flaccid paralysis. In the Emilia-Romagna region, all hospitalized patients presenting with these neurological symptoms undergo systematic laboratory testing. A case is considered as probable if the patient fulfills the clinical criteria and their serum sample shows WNV-specific IgM antibodies. A confirmed case of West Nile virus (WNV) infection requires a person to meet the clinical criteria and fulfil at least one of the following four laboratory criteria: (i) isolation of WNV from blood, urine or cerebrospinal fluid (CSF), (ii) presence of IgM antibodies in the CSF detected by ELISA, (iii) detection of WNV RNA by RT-PCR in blood, urine or CSF, or (iv) a specific antibody response (IgG and IgM) to WNV, confirmed by neutralisation assay.

Once a confirmed case of WNV infection is detected, the affected provinces implement immediate screening of all blood, hematopoietic stem cell and solid organ donations using the WNV nucleic acid amplification test (NAAT) to identify the possible presence of the virus. At the national level, all blood, tissue and solid organ donors who have traveled to an affected area must be temporarily deferred for 28 days from the date of leaving the affected area (23, 29). The same sanitary measures are implemented if WNV is detected in any other component of the integrated surveillance system, including birds.

Data on confirmed human cases of WNV infection presenting with fever (WNF) or neurological disease (WNND) were kindly provided by the Service for Collective Prevention and Public Health of the Emilia-Romagna region within the framework of the regional WNV integrated surveillance system. The data provided were anonymized and only the following were used for this study: case code, date of notification, type of disease (WNND; WNF) and province of most probable infection.

### 2.4 Timing of active surveillance on corvids

A short period of time between sampling and infection notification dates is crucial for the effectiveness of the surveillance system (30). We divided this period into three parts: (a) from sampling to laboratory registration; (b) from registration to start of testing; (c) from start of testing to notification. In Emilia-Romagna, the laboratory immediately notifies the Public Health authorities of the WNV test results by e-mail at the end of each testing day. Therefore, we have not taken into account in our analysis the period between the end of the tests and the official notification.

### 2.5 Definition of threshold for early detection

As sanitary measures are implemented at the provincial level, in the analysis we examined data for each of the eleven years (2013–2023) in the nine provinces of the region. We thus evaluated 99 different epidemic seasons. For each season, we considered the notification dates of the first human case and of the first infected bird, and determined whether the notification occurred first in birds or in humans. Maps were generated in the R environment using the tmap package (31, 32).

To calculate the best density threshold (number of birds tested/100 km^2^) for early detection of WNV in corvids, we only calculated the density of birds tested before the first human case was notified for each season. We considered the 60 seasons in which only human cases of WNV disease were notified (six cases), and the 54 seasons in which early or late detection of WNV in birds compared to humans occurred.

In our analysis, we considered an expected result to be an early detection in birds if the number of birds tested was higher than a certain threshold, or a late virus detection if the number of birds tested was lower than the threshold. For different bird density thresholds (number of corvids tested per 100 km^2^), we divided these 60 seasons in four classes: (i) seasons with early detection of WNV in corvids and the number of birds tested was over the defined threshold (EDOT); (ii) seasons with early detection in corvids but the number of birds tested was below the threshold (EDBT); (iii) seasons with late or no detection of WNV in birds and the number of birds tested was below the threshold (LDBT); (iv) seasons with late or no detection of WNV in birds and the number of birds tested was over the threshold (LDOT). Using these variables, we calculated three indices for each threshold to evaluate the performance of our surveillance system: (i) the proportion of seasons with early detection in corvids when the number of birds tested was above the threshold, calculated as follows: %EDOT = [EDOT/(EDOT+LDOT)], (ii) the proportion of seasons with late or no detection of WNV when the number of birds tested was below the threshold, calculated as: %LDBT = [LDBT/(EDBT+LDBT)], and finally (iii) the proportion of expected results, calculated as %ExpRes = [(EDOT+LDBT)/N], where N is (EDOT + LDOT + EDBT + LDBT).

## 3 Results

### 3.1 Active WNV surveillance in corvids

**Figure 2:**
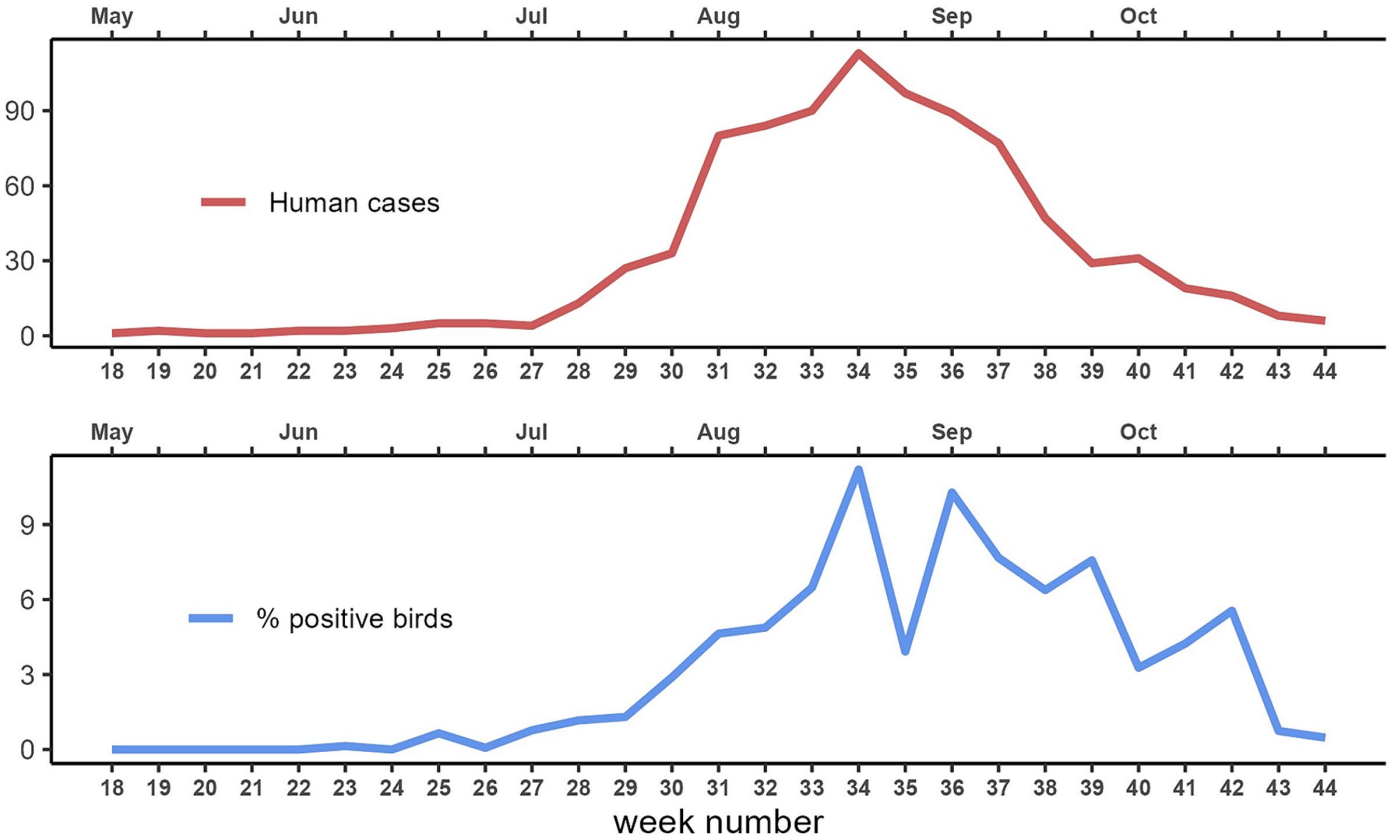
Total number of human cases of West Nile disease and average West Nile virus sample prevalence in corvids per week of notification. Emilia-Romagna region, 2013–2023.

**Figure 3:**
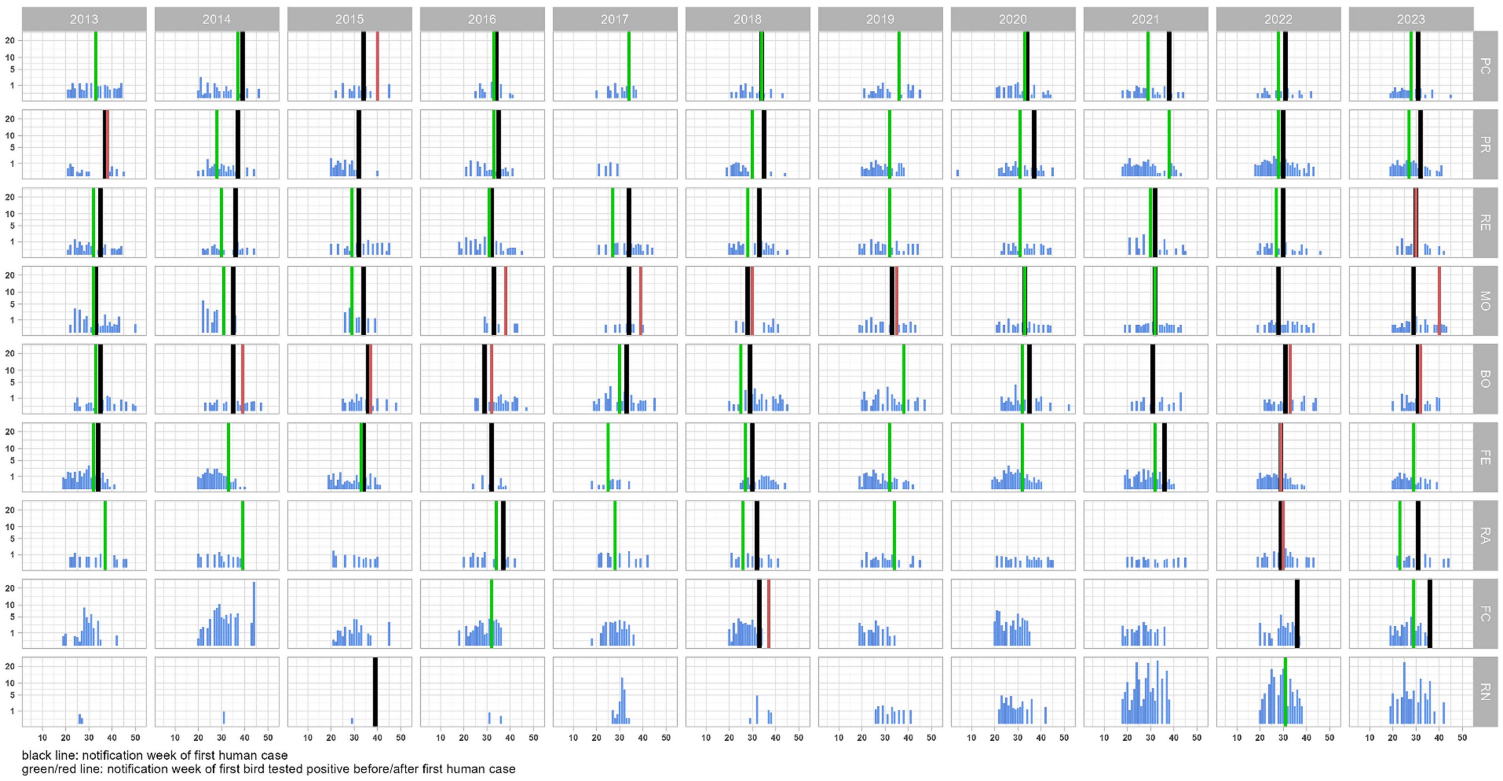
Trend of the bird density (no. of birds tested for West Nile virus per 100 km^2^) per week and province in the Emilia-Romagna region, 2013–2023. Week of notification of the first case in humans (black line) and birds (green/red lines) are highlighted.

During the eleven-year study period (2013–2023), from May to October, a total of 22,314 corvids were tested by PCR and 642 (2.9%) were found to be infected with WNV. The highest proportions of positive birds were found in magpies (489/15,632; 3.1%), and hooded crows (139/4670; 3.0%), while the level of infection was 4.4 times lower in jays (14/2012; 0.7%). Except in 2016, 2017 and 2023, when the first infected bird was notified in June (weeks 23–26), WNV was generally first detected in July (weeks 27–31), with peaks between August and September (weeks 34–39; Figure 2). In the 99 seasons evaluated, WNV was detected in birds 73 times. In 19 seasons, virus detection in birds occurred in the absence of notified human cases of WNV infection (Figure 3). Considering the 54 seasons in which the virus was notified in both corvids and humans, first detection in birds occurred in 38 seasons. As a result, WNV was first or only notified in birds in 57 seasons. On average, detection in birds occurred about three weeks earlier than in humans (Table 1; Figure 3). Further details of sampling activities and descriptive statistics for the number of birds sampled per 100 km^2^ per week in all provinces are provided in the Supplementary material.

**Table 1:** Number of days between notification of West Nile virus in humans and corvids, Emilia-Romagna region, 2013–2023.

WNV notification	N. of seasons	Mean (± SD)	Median	Q90	Min.	Max.
First in corvids	38	25.3 (± 16.7)	22	49.3	1	62
First in humans	16	21.1 (± 19.6)	15.5	40	0	75

SD, standard deviation; Q90, ninetieth quartile, Emilia-Romagna region, 2013–2023.

### 3.2 Human cases of West Nile disease

During the eleven-year study period, 469 cases of human disease due to WNV infection (125 cases of WNF, and 344 cases of WNND) were notified in the Emilia-Romagna region. In this study, we did not count the cases of WNV infection in asymptomatic donors. The highest incidence was registered in 2018 (171 cases; 38.5 cases/100,000 inhabitants). In general, human cases were first notified in July (weeks 28–29), while the peak incidence was registered in August (weeks 33–36; Figure 2). In the 99 seasons evaluated, WNV was detected in humans in 60 seasons. In six seasons, human cases were notified without virus detection in birds (Figure 3). Considering the 54 seasons in which the virus was notified in both corvids and humans, first detection in humans occurred 16 times (Table 1; Figure 3). As a result WNV was first or only notified in humans 22 times.

### 3.3 Timing of West Nile virus detection

**Figure 4:**
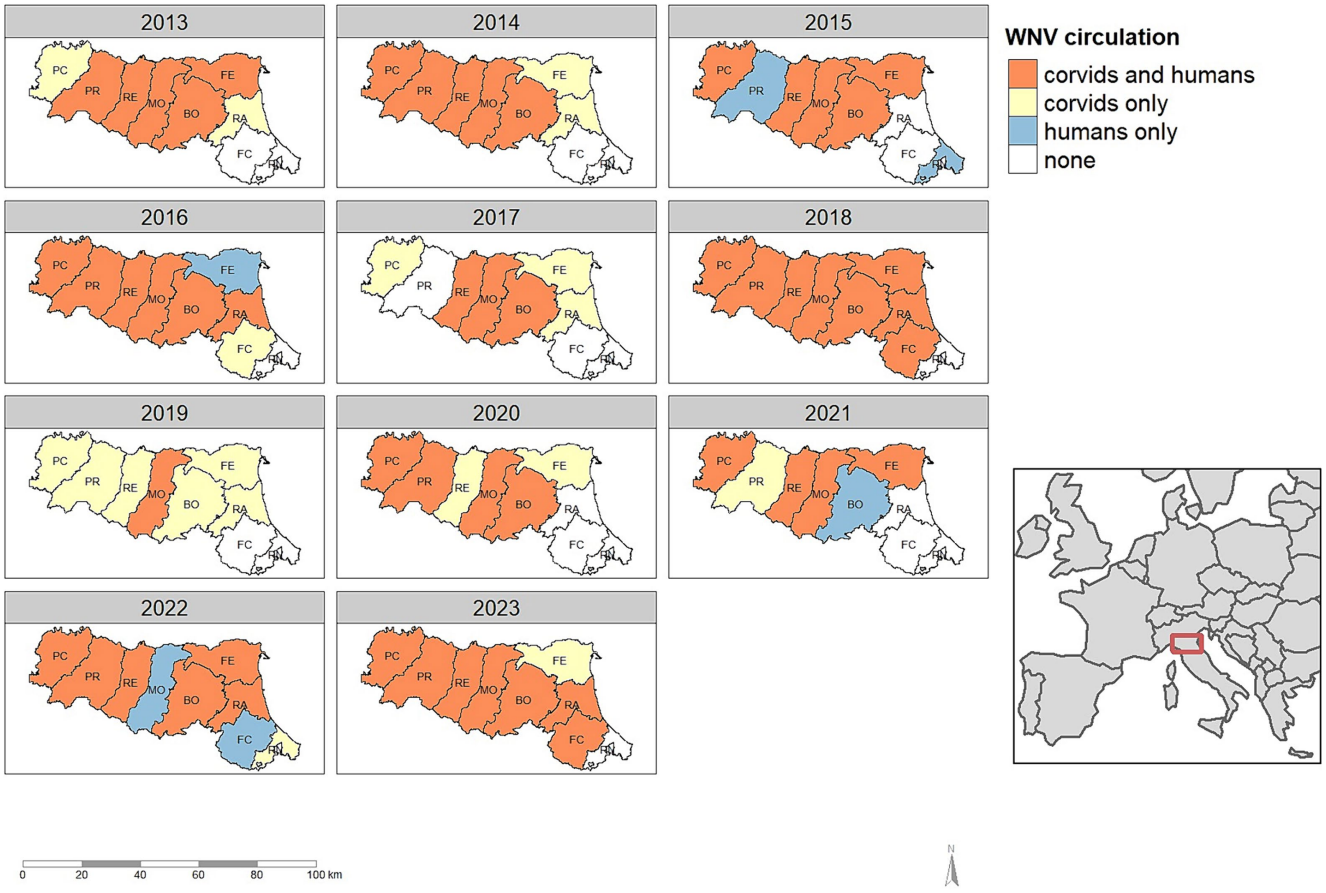
Detection of West Nile virus in humans and corvids, by province and year in the Emilia-Romagna region, 2013–2023.

During the eleven-year study period, WNV was detected in the Emilia-Romagna region every year, although not in all provinces (Figure 4). In the 99 epidemic seasons evaluated, the virus was notified 79 times: 73 times in corvids (92.4%), and 60 times in humans (75.9%).

The average time from bird sampling to notification of results was eight days and 90% of results were notified within 14 days (Table 2). An average of three days (range: 0–106) is required for hunters to transport samples from the field to the registration sites (Table 2). In Emilia-Romagna, bird and mosquito testing are carried out in the same laboratory, IZSLER, which is a multi-site laboratory with eight registration sites, but PCR testing for WNV detection is only carried out from Monday to Friday in one specific site, in the province of Reggio Emilia. On average, it takes 3.8 days (range: 0–42) to transport samples from the registration site to the laboratory for analysis (Table 2).

**Table 2:** Active West Nile virus surveillance in corvids – number of days between sampling and notification dates, Emilia-Romagna region, 2013–2023.

Period (days)	Mean (±SD)	Median	Q90	Min.	Max.
Sampling – Lab. registration	2.9 (± 7.9)	0	7	0	106
Lab. registration – Start of testing	3.8 (± 2.9)	3	7	0	42
Start of testing – Notification	1.7 (± 1.5)	1	3	0	50
Sampling – Notification	8.3 (± 8.7)	6	14	1	113

SD, standard deviation; Q90, ninetieth quartile.

### 3.4 Definition of the threshold for early detection of WNV in corvids

**Figure 5:**
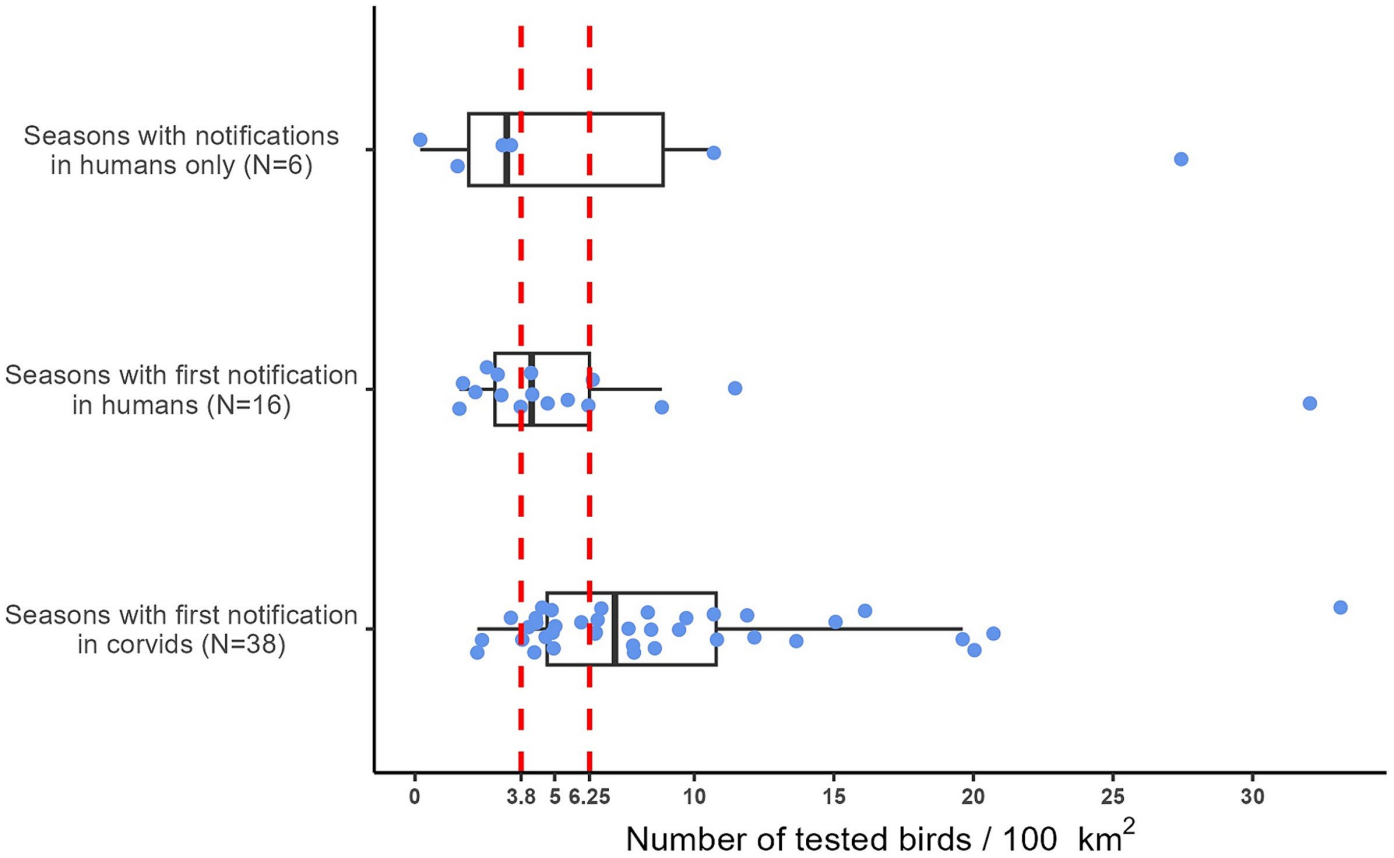
Distribution of the number of birds per 100 km^2^ tested before the notification of the first human case in the 60 seasons (blue dots) in which human cases of West Nile disease were notified. Emilia-Romagna region, 2013–2023. The two red lines represent our best threshold (3.8) and the target provided by the Italian WNV surveillance plan (6.25).

During the eleven years covered by this study, human cases of West Nile disease occurred in 60 epidemic seasons. Figure 5 shows the distribution of the number of corvids tested per 100 km^2^ prior to the notification of the first human case of West Nile disease per season. In the 38 seasons in which WNV was detected by active bird surveillance before the first human case was notified, the number of corvids tested per 100 km^2^ was higher than in the 22 seasons in which the first WNV notification occurred in humans. Notably, in 90% of the seasons with early detection in corvids, the density of birds tested per 100 km^2^ was at least 4.

**Figure 6:**
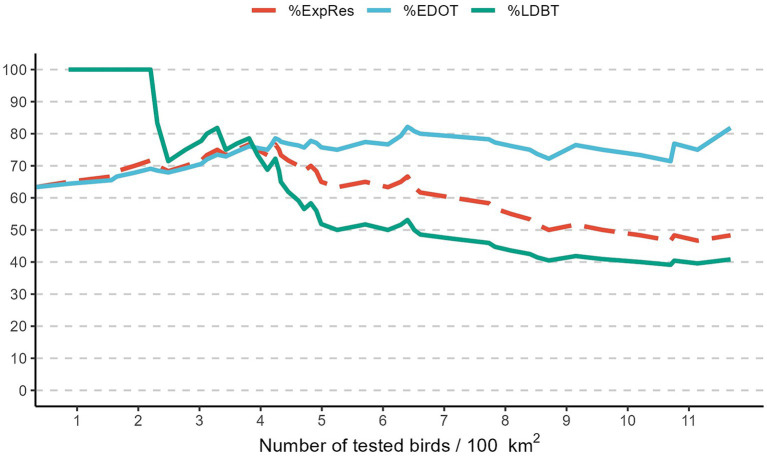
Evolution of proportion of seasons with West Nile virus early detection in corvids when the number of birds tested was above the threshold (%EDOT), proportion of seasons with late or no virus detection when the number of birds tested was below the threshold (%LDBT), and proportion of expected results (%ExpRes). Emilia-Romagna region, 2013–2023.

The highest proportion of expected results (%ExpRes) corresponds to a density between 3.8 and 4.2 birds tested per 100 km^2^ (Table 3). Starting from this values, increasing the number of birds tested per 100 km^2^ does not lead to a significant increase in %EDOT, which remains around 78%, while there is a decrease in %LDBT. This index decreases from 78.6% at 3.8 birds tested per 100 km^2^ to 51.6% when at least 6.25 birds are tested per 100 km^2^ (Figure 6).

**Table 3:** Relationship between sampling intensity and timing of virus detection in the 60 seasons with human cases of West Nile virus disease.

Threshold (corvids tested /100 km^2^)	EDOT	EDBT	LDBT	LDOT	%EDOT	%LDBT	%ExpRes
2	38	0	4	18	67.9	100.0	70.0
2.5	36	2	5	17	67.9	71.4	68.3
3.5	35	3	10	12	74.5	76.9	75.0
3.8	35	3	11	11	76.1	78.6	76.7
4.2	33	5	13	9	78.6	72.2	76.7
5	25	13	14	8	75.8	51.8	65.0
6.25	23	15	16	6	79.3	51.6	65.0
7.2	19	19	17	5	79.2	47.2	60.0
8.5	14	24	17	5	73.7	41.5	51.7
11	9	29	19	3	75.0	39.6	46.7

EDOT, Number of seasons (cases) with WNV early detection in corvids, and number of birds tested over the threshold; EDBT, Number of seasons with WNV early detection in corvids, and number of birds tested below the threshold; LDBT, Number of seasons with late or no WNV detection in corvids, and number of birds tested below the threshold; LDOT, Number of seasons with late or no WNV detection in corvids, and number of birds tested over the threshold. %EDOT = [EDOT/(EDOT + LDOT)]; %LDBT = [LDBT/(EDBT + LDBT)]; %ExpRes = [(EDOT + LDBT)/N], where *N* = 60.

## 4 Discussion

Active bird surveillance is easier to organize and implement than mosquito surveillance because it requires less expertise and is less expensive (19, 20, 33). The cost of surveillance systems is directly related to the intensity of sampling. In the Emilia-Romagna region, for the period 2009–2015, the cost of entomological surveillance, which involves activating a mosquito trap fortnightly over approximately 130 km^2^, was reported to be 2.5 times higher than the cost of bird surveillance. Mosquito surveillance is more expensive due to the need for specialized equipment, trained entomologists and time-consuming sample collection and preparation (20). Our results show that active surveillance in corvids is more sensitive than surveillance in human patients: in the 99 seasons evaluated, WNV was detected in 79 seasons, 73 times in birds (92.4%) and 60 times in human patients (75.9%).

Three parameters are required to quantify the sensitivity of an early detection surveillance system: population coverage, temporal coverage, and detection sensitivity (30). It is impossible to monitor a significant proportion of a wildlife population. Our data suggest that increasing the number of birds tested increases the probability of early detection of WNV in these animals compared to human cases. However, it is difficult to define how many animals need to be tested for early detection of the virus. In the eleven-year period, there were only 60 seasons with human cases, and the bird surveillance system is designed to sample 6.25 birds per 100 km^2^ throughout the season (May–October), so there were few seasons where this threshold was exceeded and human cases occurred. Our analysis shows that from a density of 3.8 birds per 100 km^2^ the probability of early detection of the virus remains around 79%, while increasing the number of birds tested reduces the probability of late detection of the virus compared to the occurrence of human cases of disease due to WNV infection.

In the Emilia-Romagna region, WNV circulation in birds and humans shows a clear seasonal trend, strictly related to the activity of mosquito vectors (19, 21, 22, 34). Early detection of the virus occurs in late June (weeks 25–26), the peak of incidence is around mid-August (weeks 33–36), while only rare cases in birds are notified in the second half of October (weeks 43–44). Since at our latitude winter is too cold for mosquito vectors, and totally stops WNV circulation, we monitor the virus only from May to October. As a result, we are unable to assess any long-term trends that might affect our time series.

Timely detection of the virus is essential for the activation of sanitary measures to reduce the risk of WNV transmission through blood and solid organ donations. On average in the Emilia-Romagna region, the time from bird sampling to test results is just over a week, which we consider to be excellent system performance. Since 2013, this time interval has been reduced by a few days (data not shown) and a further reduction seems difficult to achieve.

In Italy, the participation of hunters in the collection of samples is voluntary, and it is not possible to significantly reduce sample transfer and analysis times, i.e., by increasing the number of laboratories performing the tests, without a significant increase in costs.

Corvids, especially magpies, have been confirmed as valid sentinel species for WNV, and have been used in many serological surveys (13, 15). Although these species share the same environment, the proportion of infected samples in jays was about four times lower than that in hooded crows and magpies. In contrast to the other two species, the Eurasian jay (*Garrulus glandarius*) does not exhibit scavenging and gregarious behavior, which in our opinion reduces the risk of acquiring the virus through feeding on infected carcasses or direct bird-to-bird contact.

In our surveillance system, we preferred to use PCR testing for several reasons, the most important being its high specificity. Usutu virus (USUV), another flavivirus belonging to the Japanese encephalitis serocomplex, cross-reacts with West Nile virus (35, 36) and is endemic in Emilia-Romagna (37, 38). The circulation of different flaviviruses, such as WNV, USUV or Bagaza virus in the same area requires confirmation by virus neutralization or plaque reduction test of positive sera in ELISA (13, 36, 39). Unfortunately, these tests require at least three days of virus incubation (40, 41), whereas PCR can be performed within one working day. USUV infection partially protects against WNV infection (42), but several cases of co-infection have been described (38, 43, 44). Corvids experimentally infected with WNV show detectable viral genome in organs up to 17 days post infection (10–12, 45, 46). As persistent infection with WNV is a rare event in passerines (47), a positive PCR test generally indicates a recent infection, even in adult birds. Moreover, seropositivity in an adult corvid could be due to a past infection that occurred in previous years (13, 48), whereas we were interested in detecting WNV circulation in the current season. Birds that survive infection with WNV develop robust immunity (48). While a high level of immunity in the bird population is a factor limiting the circulation of the virus, it is important to remember that WNV affects many bird species and that there will always be a significant number of susceptible birds in the field (e.g., newborns, migratory birds).

In our region the intensity of WNV circulation mainly depends from two variables: (i) bird population immunity at the end of the previous year, and (ii) temperature in spring (34). We have seen that a season of intense virus circulation is usually followed by at least one season of low intensity (Supplementary Figure S2), but there are other environmental factors that change from season to season, most notably temperature, which affect the natural cycle of the virus independently of the immunity of the bird population (34). However, after a couple of seasons of weak virus circulation the immunity of the bird population decreases and the conditions for the occurrence of an intense circulation of the WNV are restored. Therefore, WNV incidence shows a multi-annual cyclicality. Given all these variables, it is almost impossible to predict the evolution of a WNV epidemic based on virus occurrence in birds alone, but this does not diminish the effective use of birds in an early warning system for West Nile virus.

In conclusion, our results show that active surveillance of corvids generally allows the detection of WNV before the onset of human cases, although occasional failures of this component of the surveillance system may occur when the number of birds tested is low. However, the other components of the integrated surveillance system in the Emilia-Romagna region, such as passive surveillance of birds or mosquito surveillance, can compensate for any shortcomings of active surveillance on corvids, because they involve other points of the WNV cycle, namely died birds with virus infection and mosquito vectors (43, 49). Based on our results, it appears that active testing of a minimum of 3.8 corvids per 100 km^2^ provides good sensitivity for this surveillance system component, but for early detection of the virus it is critical to test birds between mid-June and mid-August when mosquito activity is typically higher. This timing maximizes the chances of identifying infected birds and initiating appropriate control measures.

## Data availability statement

The dataset generated in this study can be found in Zenodo (doi: 10.5281/zenodo.10868159).

## Ethics statement

Ethical approval was not required for the study involving animals in accordance with the local legislation and institutional requirements because the authors used only birds killed by authorized hunters for other purposes.

## Author contributions

MT: Conceptualization, Methodology, Writing – original draft, Writing – review & editing. PB: Formal analysis, Methodology, Writing – review & editing. GG: Data curation, Formal analysis, Software, Writing – original draft, Writing – review & editing. GC: Investigation, Writing – original draft, Writing – review & editing. AS: Supervision, Writing – review & editing. AR: Data curation, Writing – review & editing. MC: Data curation, Formal analysis, Supervision, Writing – original draft, Writing – review & editing.
