# Supplementary material for: West Nile virus surveillance using sentinel birds: results of eleven years of testing in corvids in a region of northern Italy
Source: Front Vet Sci. 2024 May 16;11:1407271. doi: 10.3389/fvets.2024.1407271 (PMC11138491; doi:10.3389/fvets.2024.1407271)
Supplement: Supplementary file 1 [file Data_Sheet_1.docx]

Supplementary Material

West Nile virus surveillance using sentinel birds: results of eleven years of testing in corvids in a Region of Northern Italy

**Tamba, Marco^1*^, Bonilauri, Paolo^1^, GALLETTi, Giorgio^1^, Casadei, Gabriele^1^, Santi, Annalisa^1^, Rossi, Arianna^1^, Calzolari, Mattia^1^**

^1^Istituto Zooprofilattico Sperimentale della Lombardia e dell’Emilia Romagna, Brescia, Italy

* Corresponding author

Keywords: Active surveillance, Magpie (*Pica pica*), Hooded crow (*Corvus cornix*), *Corvidae*, West Nile virus

**Supplementary Tables**

Table S1. Descriptive statistics for the number of birds sampled per 100km^2^ per week in all Provinces of Emilia-Romagna region, 2013-2023

| **week** | **n** | **average** | **median** | **sd** | **min** | **max** |
| --- | --- | --- | --- | --- | --- | --- |
| 18 | 9 | 1.23 | 0.86 | 1.16 | 0.33 | 3.75 |
| 19 | 26 | 1.02 | 0.62 | 1.26 | 0.07 | 6.19 |
| 20 | 43 | 1.25 | 0.70 | 1.78 | 0.04 | 10.51 |
| 21 | 52 | 1.10 | 0.72 | 1.19 | 0.11 | 7.57 |
| 22 | 58 | 1.20 | 0.79 | 1.44 | 0.04 | 7.13 |
| 23 | 52 | 1.02 | 0.73 | 1.20 | 0.07 | 7.32 |
| 24 | 71 | 1.66 | 1.12 | 2.90 | 0.07 | 22.89 |
| 25 | 64 | 1.80 | 0.82 | 3.82 | 0.04 | 23.08 |
| 26 | 77 | 1.35 | 0.70 | 1.78 | 0.07 | 13.32 |
| 27 | 67 | 1.41 | 0.94 | 1.32 | 0.07 | 7.46 |
| 28 | 70 | 1.54 | 0.76 | 2.18 | 0.07 | 12.57 |
| 29 | 81 | 1.76 | 0.88 | 3.17 | 0.11 | 22.33 |
| 30 | 65 | 1.60 | 0.79 | 2.55 | 0.04 | 18.58 |
| 31 | 71 | 1.57 | 0.77 | 2.10 | 0.07 | 12.95 |
| 32 | 67 | 1.48 | 0.84 | 1.98 | 0.04 | 12.38 |
| 33 | 56 | 1.49 | 0.66 | 3.56 | 0.07 | 24.39 |
| 34 | 66 | 1.15 | 0.70 | 1.39 | 0.07 | 6.19 |
| 35 | 56 | 0.95 | 0.56 | 1.68 | 0.07 | 12.20 |
| 36 | 60 | 1.11 | 0.63 | 1.75 | 0.07 | 11.07 |
| 37 | 50 | 1.10 | 0.55 | 2.51 | 0.04 | 17.07 |
| 38 | 49 | 0.54 | 0.40 | 0.57 | 0.04 | 3.56 |
| 39 | 41 | 0.56 | 0.51 | 0.36 | 0.04 | 1.37 |
| 40 | 40 | 0.51 | 0.39 | 0.33 | 0.04 | 1.24 |
| 41 | 36 | 0.42 | 0.39 | 0.26 | 0.06 | 1.13 |
| 42 | 28 | 0.66 | 0.51 | 0.60 | 0.07 | 2.81 |
| 43 | 23 | 0.70 | 0.53 | 0.71 | 0.07 | 3.18 |
| 44 | 23 | 1.54 | 0.42 | 5.04 | 0.07 | 24.59 |

Table S2. Descriptive statistics for the number of birds sampled per 100km2 per week and province in Emilia-Romagna region, 2013-2023

| **Province_code** | **week** | **N_of_seasons** | **average** | **median** | **sd** | **min** | **max** |
| --- | --- | --- | --- | --- | --- | --- | --- |
| BO | 19 | 1 | 0.57 | 0.57 |  | 0.57 | 0.57 |
| BO | 20 | 4 | 0.56 | 0.60 | 0.22 | 0.27 | 0.80 |
| BO | 21 | 2 | 1.59 | 1.59 | 0.94 | 0.93 | 2.25 |
| BO | 22 | 4 | 0.57 | 0.62 | 0.23 | 0.27 | 0.80 |
| BO | 23 | 3 | 0.69 | 0.44 | 0.47 | 0.40 | 1.24 |
| BO | 24 | 8 | 0.81 | 0.53 | 0.69 | 0.18 | 1.94 |
| BO | 25 | 10 | 0.75 | 0.84 | 0.40 | 0.04 | 1.33 |
| BO | 26 | 10 | 1.08 | 0.75 | 1.06 | 0.13 | 3.71 |
| BO | 27 | 6 | 1.35 | 0.86 | 1.13 | 0.40 | 2.83 |
| BO | 28 | 5 | 0.46 | 0.53 | 0.22 | 0.22 | 0.75 |
| BO | 29 | 9 | 1.17 | 0.57 | 1.32 | 0.31 | 4.20 |
| BO | 30 | 8 | 0.67 | 0.44 | 0.49 | 0.27 | 1.77 |
| BO | 31 | 10 | 1.22 | 0.55 | 1.23 | 0.27 | 3.58 |
| BO | 32 | 8 | 1.35 | 0.91 | 1.25 | 0.04 | 3.62 |
| BO | 33 | 8 | 0.81 | 0.80 | 0.48 | 0.09 | 1.68 |
| BO | 34 | 6 | 0.76 | 0.62 | 0.53 | 0.27 | 1.72 |
| BO | 35 | 9 | 0.80 | 0.80 | 0.46 | 0.22 | 1.55 |
| BO | 36 | 7 | 0.81 | 0.75 | 0.52 | 0.27 | 1.68 |
| BO | 37 | 4 | 0.53 | 0.49 | 0.28 | 0.27 | 0.88 |
| BO | 38 | 9 | 0.64 | 0.53 | 0.44 | 0.18 | 1.37 |
| BO | 39 | 8 | 0.89 | 0.93 | 0.39 | 0.40 | 1.37 |
| BO | 40 | 8 | 0.78 | 0.84 | 0.31 | 0.35 | 1.24 |
| BO | 41 | 5 | 0.45 | 0.44 | 0.14 | 0.31 | 0.66 |
| BO | 42 | 2 | 1.44 | 1.44 | 0.28 | 1.24 | 1.64 |
| BO | 43 | 6 | 0.93 | 0.71 | 0.63 | 0.44 | 2.12 |
| BO | 44 | 6 | 0.80 | 0.82 | 0.25 | 0.44 | 1.10 |
| FC | 18 | 3 | 1.39 | 1.43 | 1.04 | 0.33 | 2.41 |
| FC | 19 | 4 | 1.65 | 1.48 | 1.30 | 0.55 | 3.07 |
| FC | 20 | 8 | 1.76 | 1.65 | 1.14 | 0.33 | 3.51 |
| FC | 21 | 7 | 2.18 | 1.65 | 2.54 | 0.11 | 7.57 |
| FC | 22 | 9 | 2.01 | 1.43 | 2.11 | 0.11 | 7.13 |
| FC | 23 | 8 | 1.44 | 1.26 | 0.71 | 0.55 | 2.52 |
| FC | 24 | 11 | 1.91 | 1.43 | 1.16 | 0.55 | 4.50 |
| FC | 25 | 10 | 2.06 | 2.31 | 1.41 | 0.33 | 4.28 |
| FC | 26 | 7 | 2.57 | 2.96 | 1.25 | 0.11 | 3.84 |
| FC | 27 | 8 | 3.38 | 3.07 | 1.82 | 1.32 | 7.46 |
| FC | 28 | 10 | 4.16 | 3.35 | 2.62 | 1.54 | 8.78 |
| FC | 29 | 11 | 3.18 | 1.65 | 2.99 | 0.77 | 10.54 |
| FC | 30 | 10 | 3.28 | 3.57 | 1.69 | 0.99 | 6.15 |
| FC | 31 | 10 | 3.12 | 2.25 | 1.45 | 1.65 | 5.93 |
| FC | 32 | 11 | 2.43 | 1.87 | 1.72 | 0.55 | 5.71 |
| FC | 33 | 8 | 2.37 | 2.09 | 1.09 | 1.10 | 3.95 |
| FC | 34 | 9 | 3.05 | 3.40 | 1.70 | 0.66 | 6.15 |
| FC | 35 | 5 | 1.51 | 1.21 | 1.25 | 0.22 | 3.18 |
| FC | 36 | 6 | 1.83 | 1.43 | 1.64 | 0.33 | 4.83 |
| FC | 37 | 4 | 2.00 | 0.60 | 2.84 | 0.55 | 6.26 |
| FC | 42 | 1 | 0.66 | 0.66 |  | 0.66 | 0.66 |
| FC | 43 | 1 | 3.18 | 3.18 |  | 3.18 | 3.18 |
| FC | 44 | 1 | 24.59 | 24.59 |  | 24.59 | 24.59 |
| FE | 18 | 1 | 0.42 | 0.42 |  | 0.42 | 0.42 |
| FE | 19 | 6 | 0.92 | 0.93 | 0.30 | 0.53 | 1.26 |
| FE | 20 | 8 | 0.94 | 0.78 | 0.74 | 0.04 | 2.36 |
| FE | 21 | 9 | 0.93 | 0.72 | 0.54 | 0.11 | 1.83 |
| FE | 22 | 8 | 0.88 | 0.93 | 0.68 | 0.04 | 1.68 |
| FE | 23 | 9 | 0.96 | 0.99 | 0.53 | 0.11 | 1.68 |
| FE | 24 | 9 | 1.41 | 1.29 | 0.76 | 0.19 | 2.63 |
| FE | 25 | 10 | 1.13 | 1.29 | 0.74 | 0.15 | 2.32 |
| FE | 26 | 10 | 1.23 | 1.10 | 0.97 | 0.30 | 3.39 |
| FE | 27 | 10 | 1.20 | 0.86 | 0.80 | 0.46 | 2.48 |
| FE | 28 | 10 | 1.02 | 0.84 | 0.69 | 0.19 | 2.55 |
| FE | 29 | 10 | 1.24 | 1.47 | 0.81 | 0.11 | 2.40 |
| FE | 30 | 10 | 1.05 | 0.65 | 1.09 | 0.04 | 3.47 |
| FE | 31 | 10 | 0.91 | 0.95 | 0.61 | 0.15 | 1.71 |
| FE | 32 | 9 | 0.70 | 0.65 | 0.34 | 0.30 | 1.29 |
| FE | 33 | 8 | 0.67 | 0.67 | 0.25 | 0.30 | 1.14 |
| FE | 34 | 10 | 0.87 | 0.84 | 0.51 | 0.15 | 1.79 |
| FE | 35 | 10 | 0.46 | 0.42 | 0.33 | 0.08 | 0.95 |
| FE | 36 | 8 | 0.66 | 0.63 | 0.52 | 0.08 | 1.64 |
| FE | 37 | 6 | 0.66 | 0.57 | 0.55 | 0.04 | 1.49 |
| FE | 38 | 8 | 0.31 | 0.27 | 0.23 | 0.04 | 0.65 |
| FE | 39 | 8 | 0.38 | 0.40 | 0.29 | 0.04 | 0.88 |
| FE | 40 | 6 | 0.24 | 0.27 | 0.14 | 0.04 | 0.38 |
| FE | 41 | 3 | 0.14 | 0.15 | 0.02 | 0.11 | 0.15 |
| FE | 42 | 1 | 0.15 | 0.15 |  | 0.15 | 0.15 |
| FE | 44 | 1 | 0.23 | 0.23 |  | 0.23 | 0.23 |
| MO | 19 | 3 | 0.38 | 0.38 | 0.00 | 0.38 | 0.38 |
| MO | 20 | 2 | 0.35 | 0.35 | 0.04 | 0.32 | 0.38 |
| MO | 21 | 3 | 0.38 | 0.38 | 0.00 | 0.38 | 0.38 |
| MO | 22 | 6 | 1.49 | 0.57 | 2.37 | 0.13 | 6.30 |
| MO | 23 | 2 | 0.54 | 0.54 | 0.49 | 0.19 | 0.89 |
| MO | 24 | 8 | 1.36 | 0.60 | 1.39 | 0.38 | 3.56 |
| MO | 25 | 3 | 0.53 | 0.38 | 0.43 | 0.19 | 1.02 |
| MO | 26 | 9 | 1.13 | 0.70 | 1.05 | 0.25 | 3.24 |
| MO | 27 | 4 | 1.14 | 0.70 | 1.11 | 0.38 | 2.80 |
| MO | 28 | 9 | 1.22 | 0.64 | 1.30 | 0.32 | 3.75 |
| MO | 29 | 8 | 1.10 | 0.76 | 0.86 | 0.25 | 2.80 |
| MO | 30 | 6 | 0.68 | 0.51 | 0.48 | 0.38 | 1.65 |
| MO | 31 | 5 | 0.95 | 0.76 | 0.78 | 0.19 | 2.10 |
| MO | 32 | 7 | 0.68 | 0.64 | 0.40 | 0.32 | 1.46 |
| MO | 33 | 4 | 0.45 | 0.45 | 0.07 | 0.38 | 0.51 |
| MO | 34 | 6 | 0.75 | 0.64 | 0.55 | 0.13 | 1.65 |
| MO | 35 | 10 | 0.66 | 0.45 | 0.45 | 0.25 | 1.53 |
| MO | 36 | 7 | 0.75 | 0.45 | 0.58 | 0.25 | 1.78 |
| MO | 37 | 4 | 0.87 | 0.54 | 0.78 | 0.38 | 2.04 |
| MO | 38 | 7 | 0.40 | 0.38 | 0.14 | 0.19 | 0.57 |
| MO | 39 | 8 | 0.67 | 0.51 | 0.38 | 0.38 | 1.27 |
| MO | 40 | 5 | 0.46 | 0.38 | 0.25 | 0.25 | 0.89 |
| MO | 41 | 6 | 0.45 | 0.45 | 0.18 | 0.19 | 0.76 |
| MO | 42 | 4 | 0.37 | 0.38 | 0.13 | 0.19 | 0.51 |
| MO | 43 | 7 | 0.58 | 0.38 | 0.46 | 0.25 | 1.59 |
| MO | 44 | 1 | 0.32 | 0.32 |  | 0.32 | 0.32 |
| PC | 18 | 1 | 0.36 | 0.36 |  | 0.36 | 0.36 |
| PC | 19 | 2 | 0.14 | 0.14 | 0.00 | 0.14 | 0.14 |
| PC | 20 | 4 | 0.36 | 0.36 | 0.06 | 0.29 | 0.43 |
| PC | 21 | 8 | 0.72 | 0.50 | 0.82 | 0.14 | 2.66 |
| PC | 22 | 6 | 0.43 | 0.40 | 0.29 | 0.07 | 0.86 |
| PC | 23 | 7 | 0.38 | 0.29 | 0.44 | 0.07 | 1.37 |
| PC | 24 | 7 | 0.67 | 0.65 | 0.44 | 0.07 | 1.44 |
| PC | 25 | 8 | 0.65 | 0.58 | 0.43 | 0.14 | 1.37 |
| PC | 26 | 7 | 0.51 | 0.36 | 0.43 | 0.14 | 1.30 |
| PC | 27 | 9 | 0.84 | 0.94 | 0.40 | 0.36 | 1.30 |
| PC | 28 | 9 | 0.58 | 0.50 | 0.32 | 0.29 | 1.37 |
| PC | 29 | 10 | 0.57 | 0.29 | 0.54 | 0.14 | 1.44 |
| PC | 30 | 4 | 0.95 | 0.90 | 0.43 | 0.50 | 1.51 |
| PC | 31 | 8 | 0.53 | 0.54 | 0.42 | 0.07 | 1.37 |
| PC | 32 | 10 | 0.55 | 0.43 | 0.46 | 0.07 | 1.58 |
| PC | 33 | 8 | 0.49 | 0.36 | 0.26 | 0.22 | 0.86 |
| PC | 34 | 8 | 0.29 | 0.22 | 0.27 | 0.07 | 0.86 |
| PC | 35 | 3 | 0.60 | 0.79 | 0.40 | 0.14 | 0.86 |
| PC | 36 | 5 | 0.95 | 0.94 | 0.58 | 0.07 | 1.66 |
| PC | 37 | 8 | 0.54 | 0.43 | 0.36 | 0.07 | 1.08 |
| PC | 38 | 4 | 0.40 | 0.32 | 0.36 | 0.07 | 0.86 |
| PC | 39 | 2 | 0.29 | 0.29 | 0.20 | 0.14 | 0.43 |
| PC | 40 | 3 | 0.79 | 1.01 | 0.57 | 0.14 | 1.22 |
| PC | 41 | 5 | 0.36 | 0.36 | 0.22 | 0.07 | 0.58 |
| PC | 42 | 5 | 0.42 | 0.29 | 0.36 | 0.07 | 1.01 |
| PC | 43 | 3 | 0.38 | 0.36 | 0.25 | 0.14 | 0.65 |
| PC | 44 | 3 | 0.55 | 0.22 | 0.71 | 0.07 | 1.37 |
| PR | 18 | 2 | 0.69 | 0.69 | 0.23 | 0.53 | 0.86 |
| PR | 19 | 4 | 0.68 | 0.66 | 0.20 | 0.46 | 0.92 |
| PR | 20 | 6 | 0.91 | 0.86 | 0.63 | 0.20 | 2.05 |
| PR | 21 | 9 | 0.84 | 0.73 | 0.54 | 0.33 | 2.05 |
| PR | 22 | 9 | 0.84 | 0.79 | 0.50 | 0.26 | 1.65 |
| PR | 23 | 9 | 0.93 | 0.73 | 0.49 | 0.26 | 1.52 |
| PR | 24 | 9 | 0.98 | 1.12 | 0.50 | 0.07 | 1.78 |
| PR | 25 | 9 | 0.57 | 0.46 | 0.38 | 0.13 | 1.12 |
| PR | 26 | 11 | 1.13 | 1.19 | 0.69 | 0.07 | 2.51 |
| PR | 27 | 9 | 0.96 | 0.86 | 0.71 | 0.20 | 2.25 |
| PR | 28 | 8 | 0.80 | 0.76 | 0.40 | 0.13 | 1.26 |
| PR | 29 | 10 | 1.02 | 1.16 | 0.58 | 0.13 | 1.72 |
| PR | 30 | 10 | 0.58 | 0.59 | 0.32 | 0.13 | 1.06 |
| PR | 31 | 8 | 0.81 | 0.63 | 0.59 | 0.26 | 1.78 |
| PR | 32 | 6 | 1.11 | 0.76 | 1.29 | 0.13 | 3.63 |
| PR | 33 | 6 | 0.50 | 0.53 | 0.25 | 0.07 | 0.73 |
| PR | 34 | 6 | 0.42 | 0.26 | 0.42 | 0.07 | 1.19 |
| PR | 35 | 5 | 0.41 | 0.46 | 0.23 | 0.13 | 0.73 |
| PR | 36 | 7 | 0.49 | 0.33 | 0.38 | 0.13 | 1.06 |
| PR | 37 | 5 | 0.55 | 0.53 | 0.31 | 0.26 | 1.06 |
| PR | 38 | 6 | 0.28 | 0.26 | 0.18 | 0.07 | 0.46 |
| PR | 39 | 6 | 0.35 | 0.20 | 0.36 | 0.13 | 1.06 |
| PR | 40 | 6 | 0.40 | 0.40 | 0.30 | 0.07 | 0.73 |
| PR | 41 | 6 | 0.52 | 0.43 | 0.32 | 0.20 | 1.06 |
| PR | 42 | 1 | 0.33 | 0.33 |  | 0.33 | 0.33 |
| PR | 43 | 2 | 0.30 | 0.30 | 0.33 | 0.07 | 0.53 |
| PR | 44 | 2 | 0.20 | 0.20 | 0.19 | 0.07 | 0.33 |
| RA | 19 | 1 | 0.70 | 0.70 |  | 0.70 | 0.70 |
| RA | 20 | 4 | 0.69 | 0.64 | 0.34 | 0.32 | 1.15 |
| RA | 21 | 6 | 1.01 | 0.96 | 0.42 | 0.58 | 1.66 |
| RA | 22 | 7 | 1.01 | 0.83 | 0.33 | 0.64 | 1.41 |
| RA | 23 | 6 | 0.60 | 0.64 | 0.15 | 0.38 | 0.77 |
| RA | 24 | 6 | 0.82 | 0.86 | 0.45 | 0.32 | 1.28 |
| RA | 25 | 5 | 0.61 | 0.51 | 0.40 | 0.26 | 1.28 |
| RA | 26 | 9 | 0.75 | 0.64 | 0.36 | 0.32 | 1.60 |
| RA | 27 | 5 | 0.65 | 0.64 | 0.28 | 0.32 | 1.02 |
| RA | 28 | 5 | 0.82 | 0.64 | 0.44 | 0.32 | 1.34 |
| RA | 29 | 8 | 0.84 | 0.67 | 0.46 | 0.32 | 1.47 |
| RA | 30 | 7 | 0.91 | 0.77 | 0.33 | 0.51 | 1.47 |
| RA | 31 | 5 | 0.67 | 0.32 | 0.85 | 0.19 | 2.18 |
| RA | 32 | 5 | 0.52 | 0.45 | 0.20 | 0.32 | 0.77 |
| RA | 33 | 7 | 0.74 | 0.58 | 0.43 | 0.13 | 1.41 |
| RA | 34 | 8 | 0.86 | 0.74 | 0.47 | 0.32 | 1.54 |
| RA | 35 | 4 | 0.69 | 0.61 | 0.33 | 0.38 | 1.15 |
| RA | 36 | 5 | 0.41 | 0.32 | 0.12 | 0.32 | 0.58 |
| RA | 37 | 8 | 0.59 | 0.54 | 0.20 | 0.45 | 1.02 |
| RA | 38 | 4 | 0.42 | 0.42 | 0.08 | 0.32 | 0.51 |
| RA | 39 | 5 | 0.59 | 0.58 | 0.12 | 0.45 | 0.77 |
| RA | 40 | 4 | 0.53 | 0.54 | 0.18 | 0.32 | 0.70 |
| RA | 41 | 4 | 0.51 | 0.54 | 0.34 | 0.06 | 0.90 |
| RA | 42 | 6 | 0.62 | 0.54 | 0.23 | 0.38 | 0.96 |
| RA | 43 | 2 | 0.61 | 0.61 | 0.05 | 0.58 | 0.64 |
| RA | 44 | 2 | 0.42 | 0.42 | 0.14 | 0.32 | 0.51 |
| RE | 18 | 1 | 0.98 | 0.98 |  | 0.98 | 0.98 |
| RE | 19 | 3 | 0.33 | 0.21 | 0.33 | 0.07 | 0.70 |
| RE | 20 | 4 | 0.61 | 0.74 | 0.27 | 0.21 | 0.77 |
| RE | 21 | 6 | 0.88 | 0.63 | 0.73 | 0.14 | 1.82 |
| RE | 22 | 5 | 0.45 | 0.42 | 0.19 | 0.21 | 0.70 |
| RE | 23 | 6 | 0.50 | 0.42 | 0.45 | 0.07 | 1.19 |
| RE | 24 | 9 | 1.00 | 1.05 | 0.57 | 0.21 | 1.68 |
| RE | 25 | 5 | 0.64 | 0.49 | 0.51 | 0.21 | 1.47 |
| RE | 26 | 8 | 0.54 | 0.56 | 0.30 | 0.14 | 1.05 |
| RE | 27 | 10 | 0.81 | 0.67 | 0.65 | 0.07 | 2.45 |
| RE | 28 | 9 | 0.44 | 0.21 | 0.39 | 0.07 | 1.05 |
| RE | 29 | 7 | 0.74 | 0.56 | 0.58 | 0.14 | 1.96 |
| RE | 30 | 6 | 1.03 | 0.77 | 0.82 | 0.07 | 2.10 |
| RE | 31 | 7 | 0.67 | 0.77 | 0.29 | 0.14 | 0.98 |
| RE | 32 | 5 | 0.67 | 0.77 | 0.24 | 0.42 | 0.91 |
| RE | 33 | 3 | 0.19 | 0.21 | 0.11 | 0.07 | 0.28 |
| RE | 34 | 8 | 0.60 | 0.70 | 0.42 | 0.14 | 1.12 |
| RE | 35 | 7 | 0.41 | 0.21 | 0.39 | 0.07 | 1.12 |
| RE | 36 | 10 | 0.57 | 0.32 | 0.48 | 0.07 | 1.40 |
| RE | 37 | 8 | 0.46 | 0.53 | 0.33 | 0.07 | 0.98 |
| RE | 38 | 6 | 0.41 | 0.42 | 0.30 | 0.07 | 0.70 |
| RE | 39 | 4 | 0.42 | 0.39 | 0.25 | 0.21 | 0.70 |
| RE | 40 | 8 | 0.43 | 0.35 | 0.28 | 0.07 | 0.84 |
| RE | 41 | 6 | 0.26 | 0.25 | 0.14 | 0.07 | 0.49 |
| RE | 42 | 6 | 0.44 | 0.46 | 0.32 | 0.07 | 0.84 |
| RE | 43 | 2 | 0.21 | 0.21 | 0.00 | 0.21 | 0.21 |
| RE | 44 | 7 | 0.36 | 0.28 | 0.22 | 0.07 | 0.70 |
| RN | 18 | 1 | 3.75 | 3.75 |  | 3.75 | 3.75 |
| RN | 19 | 2 | 4.03 | 4.03 | 3.05 | 1.88 | 6.19 |
| RN | 20 | 3 | 5.75 | 5.63 | 4.69 | 1.13 | 10.51 |
| RN | 21 | 2 | 2.44 | 2.44 | 1.86 | 1.13 | 3.75 |
| RN | 22 | 4 | 3.52 | 3.56 | 1.77 | 1.31 | 5.63 |
| RN | 23 | 2 | 6.10 | 6.10 | 1.72 | 4.88 | 7.32 |
| RN | 24 | 4 | 9.85 | 6.94 | 9.18 | 2.63 | 22.89 |
| RN | 25 | 4 | 14.35 | 15.10 | 8.19 | 4.13 | 23.08 |
| RN | 26 | 6 | 4.25 | 1.97 | 4.89 | 0.56 | 13.32 |
| RN | 27 | 6 | 2.56 | 2.81 | 1.76 | 0.19 | 4.69 |
| RN | 28 | 5 | 4.62 | 3.75 | 4.84 | 0.19 | 12.57 |
| RN | 29 | 8 | 5.98 | 1.69 | 8.22 | 0.19 | 22.33 |
| RN | 30 | 4 | 7.32 | 4.60 | 7.76 | 1.50 | 18.58 |
| RN | 31 | 8 | 4.41 | 2.25 | 4.57 | 0.56 | 12.95 |
| RN | 32 | 6 | 5.41 | 4.03 | 3.88 | 1.88 | 12.38 |
| RN | 33 | 4 | 9.62 | 6.94 | 11.21 | 0.19 | 24.39 |
| RN | 34 | 5 | 2.85 | 1.31 | 2.83 | 0.19 | 6.19 |
| RN | 35 | 3 | 5.88 | 3.56 | 5.54 | 1.88 | 12.20 |
| RN | 36 | 5 | 4.69 | 3.75 | 4.44 | 0.38 | 11.07 |
| RN | 37 | 3 | 7.38 | 3.94 | 8.51 | 1.13 | 17.07 |
| RN | 38 | 5 | 1.65 | 1.13 | 1.15 | 0.75 | 3.56 |
| RN | 41 | 1 | 1.13 | 1.13 |  | 1.13 | 1.13 |
| RN | 42 | 2 | 2.25 | 2.25 | 0.80 | 1.69 | 2.81 |

**Supplementary Figures**


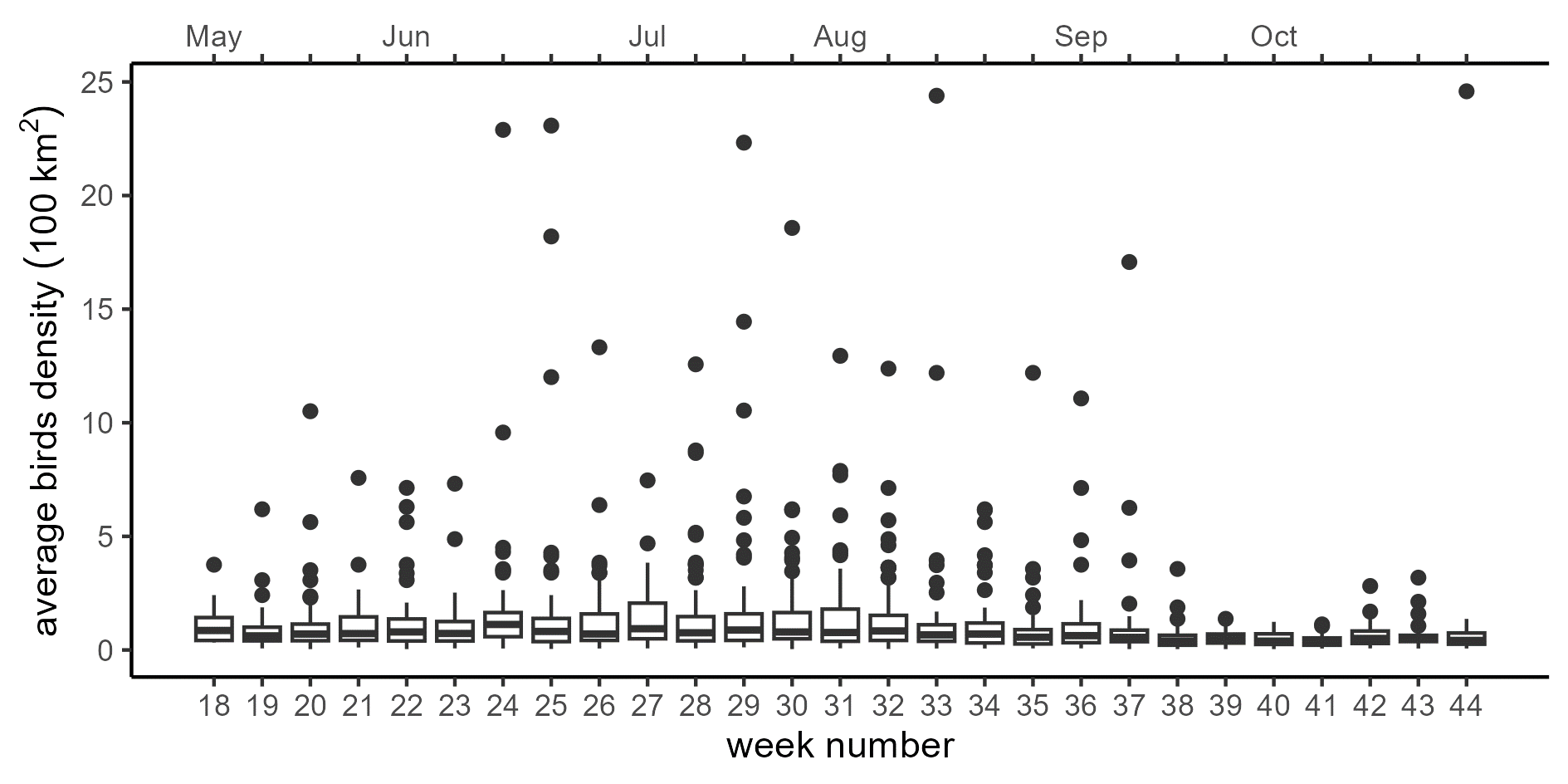


Figure S1. Trend of bird density (total number of birds sampled 100 km^2^) per week over the sampling period. Emilia-Romagna region, 2013-2023


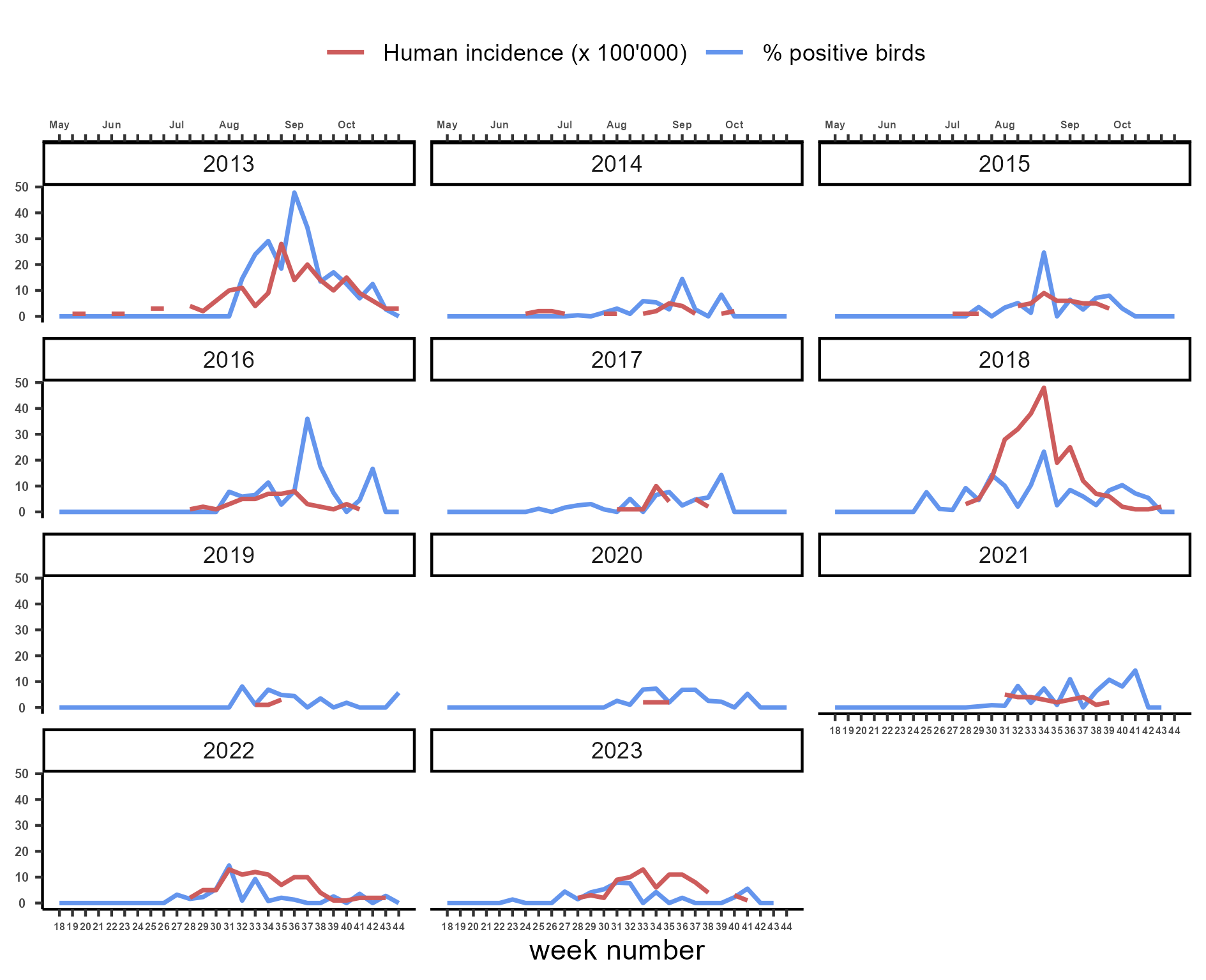


Figure S2. Trend of incidence (cases per 100,000 inhabitants) of West Nile disease in humans and sample prevalence (PCR positive per 100 of birds tested) of West Nile virus per year in the Emilia-Romagna region, 2013-2023.
